# A CRISPR-Cas13a Based Strategy That Tracks and Degrades Toxic RNA in Myotonic Dystrophy Type 1

**DOI:** 10.3389/fgene.2020.594576

**Published:** 2020-12-10

**Authors:** Nan Zhang, Brittani Bewick, Guangbin Xia, Denis Furling, Tetsuo Ashizawa

**Affiliations:** ^1^Department of Neurology, Houston Methodist Research Institute, Houston, TX, United States; ^2^Indiana University School of Medicine, Fort Wayne, IN, United States; ^3^Institut National de la Sante et de la Recherche Medicale (INSERM), Centre de Recherche en Myologie (CRM), Association Institut de Myologie, Sorbonne Université, Paris, France

**Keywords:** myotonic dystrophy, neurodegeneration, CRISPR-Cas13a, RNA targeting, stress granule

## Abstract

Cas13a, an effector of type VI CRISPR-Cas systems, is an RNA guided RNase with multiplexing and therapeutic potential. This study employs the *Leptotrichia shahii* (*Lsh*) Cas13a and a repeat-based CRISPR RNA (crRNA) to track and eliminate toxic RNA aggregates in myotonic dystrophy type 1 (DM1) – a neuromuscular disease caused by CTG expansion in the *DMPK* gene. We demonstrate that *Lsh*Cas13a cleaves CUG repeat RNA in biochemical assays and reduces toxic RNA load in patient-derived myoblasts. As a result, *Lsh*Cas13a reverses the characteristic adult-to-embryonic missplicing events in several key genes that contribute to DM1 phenotype. The deactivated *Lsh*Cas13a can further be repurposed to track RNA-rich organelles within cells. Our data highlights the reprogrammability of *Lsh*Cas13a and the possible use of Cas13a to target expanded repeat sequences in microsatellite expansion diseases.

## Introduction

Recent advances in genome editing have become increasingly important for addressing monogenic disorders. Microsatellite expansion diseases are a group of more than 30 incurable neurological and neuromuscular disorders that are caused by the expansion of 3–10 nucleotide repeats in the residing gene. In affected individuals, repeat expansions can occur in both coding and non-coding regions, resulting in toxic protein gain-of-function and RNA gain-of-function, respectively. Large repeat size often correlates positively with intergenerational instability and disease severity. When repeat expansions are located in non-coding regions, diseases such as myotonic dystrophy types 1 and 2 (DM1/2), *C9orf72-*amyotrophic lateral sclerosis/frontotemporal dementia (*C9orf72*-ALS/FTD), fragile-X tremor ataxia syndrome (FXTAS), spinocerebellar ataxias types 8, 10, 12, 31, and 36 (SCA 8/10/12/31/36), Huntington’s disease-like 2 (HDL2), and Fuch’s endothelial corneal dystrophy (FECD) occur (Mohan et al., 2014; Rohilla and Gagnon, 2017; Zhang and Ashizawa, 2017).

DM1 is the most prevalent adult-onset muscular dystrophy for which there is currently no treatment. This multisystemic disease affects approximately 1 in 8,000 individuals and is caused by a CTG repeat expansion within the 3′-untranslated region (3′-UTR) of the *DMPK* gene (Brook et al., 1992; Mahadevan et al., 1992; Thornton, 2014). The widely accepted pathogenic mechanism suggests that the expanded CUG repeat is retained in the nucleus and forms RNA foci, sequestering the MBNL (Muscleblind) family of regulators of alternative splicing and inducing upregulation of CELF1 (CUG-BP and Elav-like family member 1, another regulator of alternative splicing) through PKC (protein kinase C)-mediated phosphorylation, and together leading to a cascade of downstream missplicing events (Jiang et al., 2004; Kuyumcu-Martinez et al., 2007; Chau and Kalsotra, 2015). Other than spliceopathy, pathomechanisms involving transcription, mRNA transport and localization, polyadenylation, conventional and repeat-associated non-AUG (RAN) translation, and microRNA expression may also contribute to disease etiology to different extents (Ebralidze et al., 2004; Krol et al., 2007; Du et al., 2010; Huichalaf et al., 2010; Rau et al., 2011; Fernandez-Costa et al., 2013; Timchenko, 2013; Batra et al., 2014; Kim et al., 2014; Goodwin et al., 2015; Gourdon and Meola, 2017; Zhang and Ashizawa, 2017; Cappella et al., 2018; Cerro-Herreros et al., 2018).

Several therapeutic approaches have been designed to treat DM1 and can be broadly grouped as (Nelson et al., 2017): (1) targeting DNA repeats [e.g., CRISPR-Cas9 with CTG-flanking guide sgRNAs in DM1 myoblasts, induced pluripotent stem cells and myogenic cells (Provenzano et al., 2017; van Agtmaal et al., 2017; Dastidar et al., 2018; Wang et al., 2018) and TALEN in yeast (Richard et al., 2014)], (2) degrading RNA repeats [e.g., synthetic siRNAs (Sobczak et al., 2013), chemically modified antisense oligonucleotide (ASO) gapmers (Wheeler et al., 2012; Jauvin et al., 2017; Manning et al., 2017), bleomycin-cleavage modules (Rzuczek et al., 2017), deactivated Cas9 (dCas9) conjugated to a PIN domain (Batra et al., 2017), engineered human U7-snRNA-CUG (Francois et al., 2011), hammerhead ribozymes (Langlois et al., 2003), and small RNase mimics (Nguyen et al., 2015)], (3) dissociating CUG-MBNL interactions [e.g., small molecules (Gareiss et al., 2008; Warf et al., 2009; Childs-Disney et al., 2013; Nguyen et al., 2015; Rzuczek et al., 2015), miniPEG-γ peptides with terminal pyrenees (Hsieh et al., 2018), and steric-blocking morpholino ASOs (Wheeler et al., 2009)], and (4) small molecules that target cellular pathways independent of CUG expansions (Lopez-Morato et al., 2018). Furthermore, dCas9 has been used to impede CUG repeat transcription through steric blockage (Pinto et al., 2017), and the RIBOTAC technology coupled with RNase L could potentially be adopted to target repeat expansions in DM1 (Costales et al., 2018). Given that DNA editing using CRISPR-Cas9 is hindered by large deletions and rearrangements (Kosicki et al., 2018), p53-mediated DNA damage response (Haapaniemi et al., 2018; Ihry et al., 2018), and RNA polymerase movement (Clarke et al., 2018), an RNA-based platform might serve as an alternative therapeutic strategy. Synthetic siRNAs typically do not target nuclear repeat RNA efficiently, possibly due to the preferential distribution of the RISC (RNA-induced silencing complex) in the cytoplasm. ASOs, when administered peripherally for muscle degeneration, are often not taken up by local tissues. For instance, the IONIS-DMPK_RX_ human trial for DM1 patients did not reach the desired endpoints^1^. In addition, chemically modified ASOs with high-affinity nucleic analogs [e.g., locked nucleic acid (LNA) or constrained ethyl (cEt) nucleic acid] can induce RNase H1-dependent hepatotoxicity and apoptosis in both mice and cell models (Kakiuchi-Kiyota et al., 2014; Burel et al., 2016; Kasuya et al., 2016). Thus, an alternative reprogrammable RNA-based strategy may provide important therapeutic value in treating DM1, and by extension other microsatellite expansion diseases. However, that is not to say that the alternative reprogrammable RNA-based strategy does not face its own mechanistic and delivery challenges.

The recently discovered CRISPR-Cas13a protein belongs to the type VI-A CRISPR-Cas system and is an RNA-guided dual-functional RNase (East-Seletsky et al., 2016; Liu et al., 2017; Shmakov et al., 2017; Smargon et al., 2017; Konermann et al., 2018; Yan et al., 2018). Cas13a from *Leptotrichia shahii* (*Lsh*) utilizes a guide CRISPR RNA (crRNA) containing a 28-nucleotide (nt) spacer sequence to cleave its target RNA. The design of crRNA requires the 3′-protospacer flanking sequence (PFS) to end with A, U, or C (Abudayyeh et al., 2016). The N-terminal domain (NTD) and Helical 1 of *Lsh*Cas13a are responsible for crRNA maturation, while the two C-terminal HEPN domains are responsible for target RNA cleavage preferentially at uracils (Abudayyeh et al., 2016; Liu et al., 2017). This dual-functional nature enables multiplexing of several spacers in a single precursor crRNA (Gootenberg et al., 2018). The CRISPR-Cas13a system holds great potential for scalable RNA editing applications with reprogrammable crRNA. Evidently since its discovery, the system has been used for attomolar RNA detection by taking advantage of its collateral RNA cleavage (East-Seletsky et al., 2017; Gootenberg et al., 2018; Myhrvold et al., 2018), RNA knockdown in *Escherichia coli* and mammalian and plant cells (Abudayyeh et al., 2017; Adams et al., 2017; Zhao et al., 2018), and base editing with a deactivated Cas13a conjugated to ADAR2 (adenosine deaminase acting on RNA type 2) (Cox et al., 2017; Jing et al., 2018). Other sub-classes of Cas13 proteins (Cas13b, c and d) have been identified (Smargon et al., 2017; Konermann et al., 2018; Yan et al., 2018).

In this study, we repurposed the human codon optimized *Lsh*Cas13a protein to track and degrade toxic RNA with a repeat-based crRNA. We observed CUG repeat cleavage biochemically and in a DM1 patient-derived myoblast cell line through lentiviral delivery. In the transduced DM1 myoblast cell line, we observed CUG RNA foci reduction, *DMPK* RNA knockdown and near-complete reversal of DM1-associated splicing defects in the tested genes.

## Results

### Engineering a Deactivated *Lsh*Cas13a Tracker to Visualize CUG Repeat RNA Foci in the Nucleus

**FIGURE 1:**
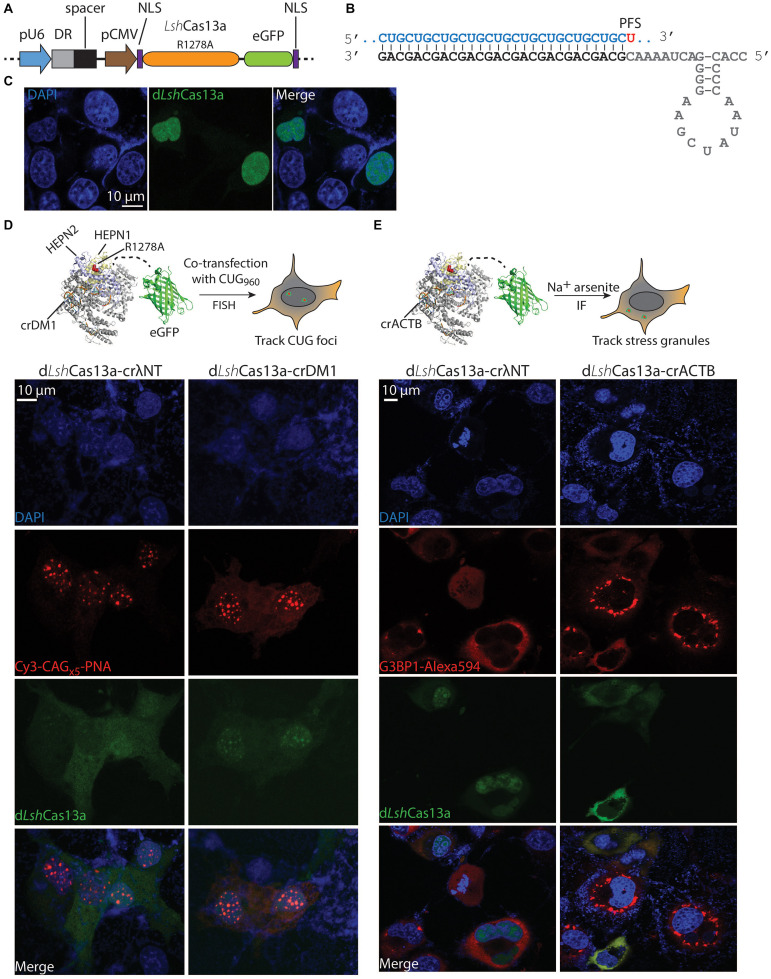
Tracking the expanded CUG RNA foci and stress granules in COS-M6 cells. **(A)** Mammalian expression vector pX330 encoding crRNA under a pU6 promoter and d*Lsh*Cas13a (*Lsh*Cas13a_R1278A_-eGFP) under a pCMV promoter. The crRNA contains the direct repeat hairpin (DR, gray box) and the spacer (black box). The d*Lsh*Cas13a is flanked by an N-terminal nucleoplasmin NLS and a C-terminal c-Myc NLS (Ray et al., 2015). **(B)** Structure of the crDM1 with DR sequence highlighted in gray, spacer in black and target CUG repeats in blue. The 3′-PFS uracil is highlighted in red. **(C)** Without the expression of crRNA, d*Lsh*Cas13a primarily localizes in the nucleus. **(D)** Comparison of non-targeting (crλNT) and CUG-targeting (crDM1) d*Lsh*Cas13a tracking nuclear CUG foci in COS-M6 cells. The expression of the interrupted CUG_x960_ repeats is driven by a CMV promoter in DT960 plasmid. **(E)** Comparison of non-targeting (crλNT) and *ACTB*-targeting (crACTB) d*Lsh*Cas13a tracking cytoplasmic stress granules in COS-M6 cells. The stress condition was induced by adding 200 μM sodium arsenite for 1 h 30 min before fixation. Stress granules are labeled with anti-G3BP1 Alexa 594 antibodies by immunofluorescence (IF).

The R1278A mutant of *Lsh*Cas13a was previously reported to have lost RNA cleavage activity but retained high RNA binding affinity [>6-fold tighter than wildtype protein (Abudayyeh et al., 2016)]. One application of this mutant is to be engineered as an RNA tracker in cells. The formation of nuclear CUG RNA foci is a hallmark of DM1 cells; currently the means to detect such foci are limited. We hypothesize that the deactivated *Lsh*Cas13a_R1278A_ mutant tethered to an eGFP (enhanced green fluorescent protein, hereafter the conjugate is named d*Lsh*Cas13a) could serve as a tool to visualize RNA foci that cause DM1. Under such premises, the *Lsh*Cas13a_R1278A_-eGFP sequence was first cloned into the pX330 backbone along with two terminal nuclear localization signals (NLSs), the pU6-driven direct repeat (DR) hairpin, and two inverted type II restriction sites for spacer insertion (Figures 1A,B). Dual NLS-tags have been extensively used in RNA targeting as well as DNA editing Cas systems (Cong et al., 2013; Nelles et al., 2016; Konermann et al., 2018). Given the primary goal of this study is to track and eliminate nuclear CUG foci, we decided to adopt the dual NLS labeling system. Without the presence of crRNA, the dual NLS-labeled d*Lsh*Cas13a largely resides in the nucleus of COS-M6 cells (Figure 1C). Subsequently to track DM1 repeats, a 28 nt spacer complementary to CUG RNA was designed with a 3′-PFS of uracil (hereafter named crDM1, Figure 1B). To create the expanded CUG RNA foci, the DT960 plasmid encoding human *DMPK* exons 11–15 with 960 interrupted CTG repeats was co-transfected with the eGFP-d*Lsh*Cas13a-crDM1 tracker plasmid into COS-M6 cells (Figure 1D). Fluorescence *in situ* hybridization (FISH) detected punctate colocalization between CUG RNA foci and the CUG-targeting d*Lsh*Cas13a (Figure 1D). In contrast, the non-targeting d*Lsh*Cas13a with a 28nt spacer against a lambda phage sequence (crλNT) showed significantly less colocalization as calculated by Pearson’s correlation (*p* = 0.0009, Supplementary Figure 1). The use of Cos-M6 cells overexpresses expanded CUG RNA to mimic and encourage RNA foci formation as seen in patient cells, however, Cas13a proteins are also overexpressed that could potentially form macromolecular aggregates. To date, most of the published Cas-based RNA tracking systems have been established in either HEK293FT or U2OS cells (Nelles et al., 2016; Abudayyeh et al., 2017; Konermann et al., 2018) – both are overexpressing cell lines. Such Cas protein aggregates have not been reported or had impacts on cell proliferation, global RNA size distribution and RNA integrity. We conclude that the d*Lsh*Cas13a tracker enables direct visualization of the expanded CUG RNA foci in COS-M6 cells.

### d*Lsh*Cas13a Can Be Reprogrammed to Visualize Stress Granules in the Cytoplasm

Stress granules are cytoplasmic, translationally silent RNA-protein aggregates that resemble the expanded CUG RNA foci; their role in neurodegeneration has been increasingly recognized (Li et al., 2013; Zhang and Ashizawa, 2017; Benarroch, 2018). Many polyadenylated mRNAs (e.g., *ACTB* mRNA) and protein markers (e.g., Ras GTPase-activating protein-binding protein 1 – G3BP1) are known to localize to stress granules under stress conditions (Tourriere et al., 2003; Unsworth et al., 2010; Matsuki et al., 2013; Bley et al., 2015). To test the reprogrammability of d*Lsh*Cas13a to track stress granules in COS-M6 cells, we generated a crRNA targeting the 3′-UTR of the *ACTB* gene (crACTB, Figure 1E) into the tracker plasmid. After treating COS-M6 cells with sodium arsenite to induce stress (Nelles et al., 2016; Wheeler et al., 2016; Abudayyeh et al., 2017), we observed significant overlap between the *ACTB*-targeting tracker and the antibody-labeled G3BP1 stress granule marker, in comparison to the non-targeting d*Lsh*Cas13a (*p* = 0.0069, Figure 1E and Supplementary Figure 2). Our data are in line with existing literature where crRNA may be a primary drive of target recognition and Cas protein complex localization, even though the d*Lsh*Cas13a protein is dual NLS-tagged. Consistently, another Cas13a protein that is predominantly nucleus-localized translocates to the cytoplasm when complexed with a crRNA targeting *ACTB* mRNA in the stress granule; while dual NLS-tagged RCas9 translocates to the cytoplasm when complexed with a *GAPDH*-targeting sgRNA (Nelles et al., 2016; Abudayyeh et al., 2017). Overall, the above data demonstrate the versatility of d*Lsh*Cas13a-eGFP in tracking nuclear and cytoplasmic RNA targets. By inference, we suggest that d*Lsh*Cas13a equipped with appropriate crRNA can be used to track other RNA-rich organelles in the cell.

### *Lsh*Cas13a Promotes CUG Repeat Cleavage in Biochemical Assays and in Patient-Derived Myoblasts

**FIGURE 2:**
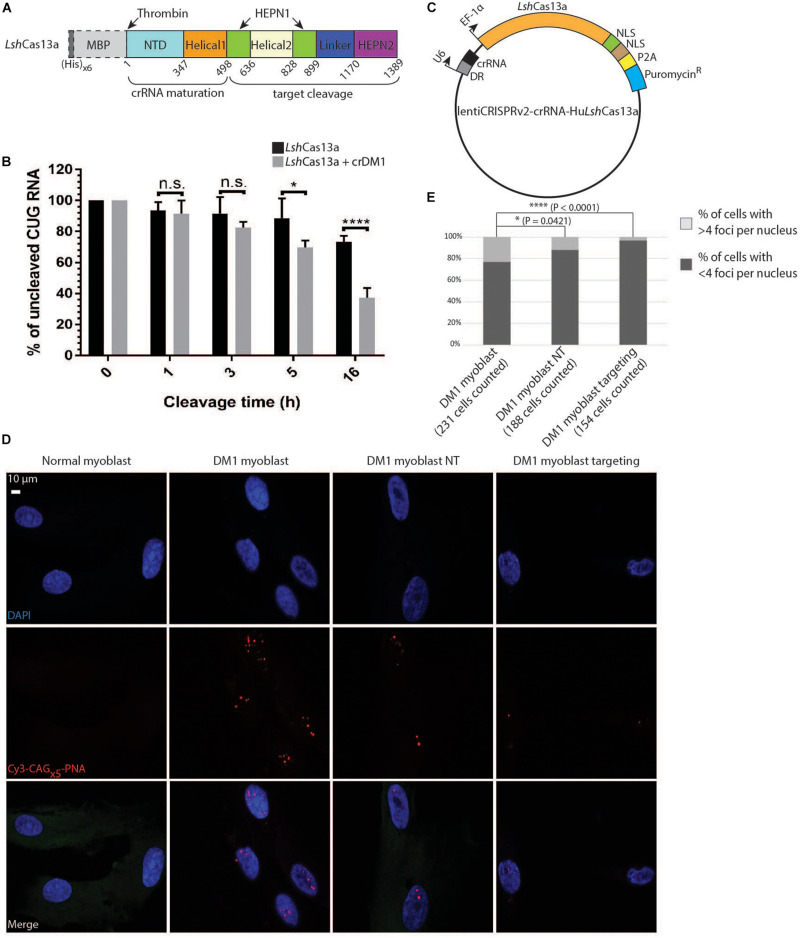
Degradation of CUG repeats in biochemical assays and in DM1 patient-derived myoblasts. **(A)** Domain organization of *Lsh*Cas13a purified in *E. coli* (not drawn to scale). NTD stands for N-terminal domain. The (His)_x6_ and maltose binding protein (MBP) tags were removed after purification by thrombin cleavage (dotted box). **(B)** Cleavage assay of CUG_x30_ RNA targets using purified *Lsh*Cas13a with or without *in vitro* transcribed crDM1. The RNA cleavage products were separated on a 10% TBE-Urea gel and quantified by Fiji ImageJ. The amount of uncut RNA at each time point was expressed as a percentage of the quantity at 0 h (100%). Error bars were calculated from four independent experiments using the same protein and RNA preparations. **(C)** Lentiviral construct for *Lsh*Cas13a-crRNA delivery. The EF-1α-driven *Lsh*Cas13a was conjugated to a nucleoplasmin NLS, an SV40 NLS, a P2A cleavage signal and an in-frame puromycin-resistant (puromycin^R^) gene. **(D)** Comparison of CUG RNA foci in normal myoblasts, untreated DM1 myoblasts, non-targeted DM1 myoblasts (DM1 myoblast NT), and CUG-targeted DM1 myoblasts (DM1 myoblast targeting) by FISH. **(E)** Quantification of foci counts of cell lines created in panel **(D)**. The number of cells used for quantification was gathered from two experiments each with two technical replicates.

To investigate if *Lsh*Cas13a can cleave CUG repeats biochemically, we expressed and purified the protein in *E. coli* (Figure 2A and Supplementary Figures 3A,B). To obtain the target CUG repeats, 30 CTG repeats were generated by self-priming PCR, cloned into the pcDNA3.1/hygro(+) vector, linearized and *in vitro* transcribed under a T7 promoter. In the presence of crDM1, we observed ≈58% total target cleavage in 16 h (Figure 2B). This is in marked contrast to the background cleavage as observed in the reaction without crDM1 (≈30% of total RNA target cleaved, Figure 2B). Interestingly, multiple target-guide RNA duplexes were observed under denaturing conditions (7 M Urea and 30% formamide at 95°C, Supplementary Figure 3C). *Lsh*Cas13a seems to be able to overcome the stability of these CUG-CAG duplexes and achieve efficient cleavage. Overall, the above data suggest that *Lsh*Cas13a promotes CUG repeat RNA cleavage in biochemical assays.

We next examined the ability of *Lsh*Cas13a to degrade endogenous CUG repeats in a DM1 patient derived myoblast cell line (>176 CTG repeats). To ensure that all tested DM1 myoblasts express *Lsh*Cas13a and crRNA, we constructed the lentiCRISPR v2-crRNA-*Lsh*Cas13a vector with type II restriction sites for spacer insertion, and subsequently used the constructs for lentiviral transduction into the cell line (Figure 2C). Puromycin treatment of the transduced cell lines (5 days at 2 μg/ml) selected for cells expressing the puromycin-resistant gene in frame with *Lsh*Cas13a and a P2A cleavage signal (Figure 2C). By performing FISH, we detected a significant reduction of RNA foci post transduction of the CUG-targeting *Lsh*Cas13a (Figures 2D,E). Approximately 3% of DM1 myoblasts transduced with CUG-targeting *Lsh*Cas13a contained >4 foci per cell, as opposed to 23% of the non-treated cells and 12% of cells transduced with non-targeting *Lsh*Cas13a (Figure 2E). Taken together, the above data indicate that *Lsh*Cas13a can indeed cleave the expanded CUG RNA foci in DM1 patient derived myoblasts.

### *Lsh*Cas13a Rescues Splicing Defects in DM1 Patient-Derived Myoblasts

**FIGURE 3:**
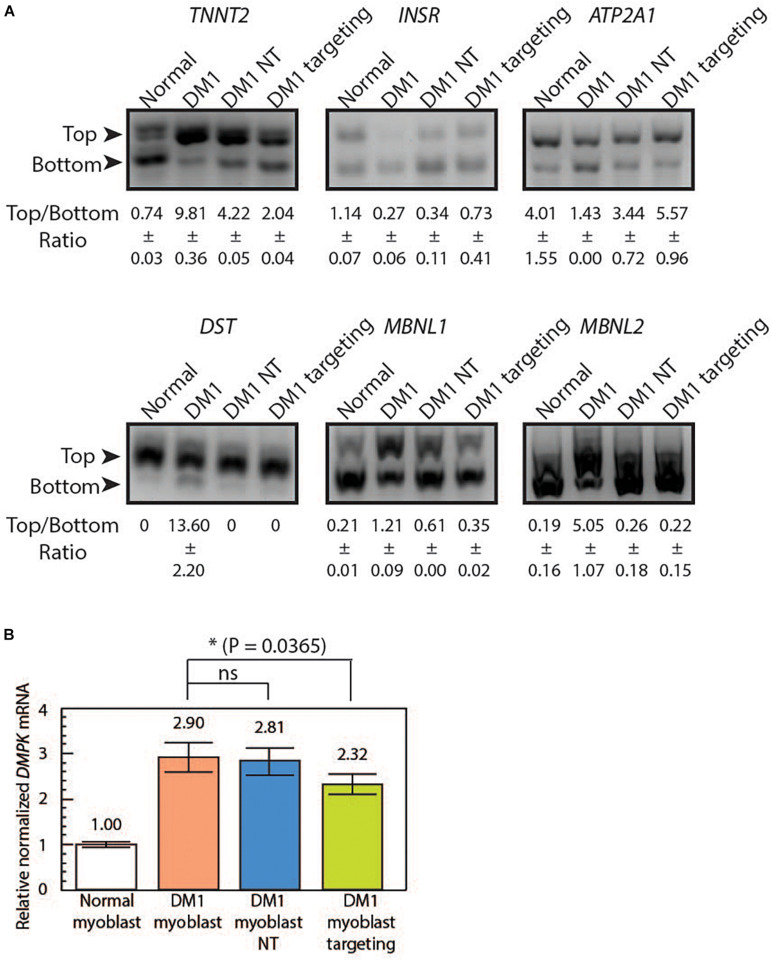
*Lsh*Cas13a reverses downstream missplicing events and knocks down *DMPK* mRNA quantity in transduced DM1 myoblasts. **(A)** Splicing patterns from six MBNL1-regulated genes were analyzed in normal myoblasts, untreated DM1 myoblasts, DM1 myoblasts NT and DM1 myoblasts targeting. The ratios of top and bottom bands (as indicated by arrows) were quantified with standard deviation (±SD) in Fiji ImagJ from two separate experiments. **(B)** The *DMPK* mRNA level was measured using qRT-PCR from the four cell lines. The relative *DMPK* mRNA level from each cell line was normalized to that of the normal myoblast (white bar). Three biological replicates each with three technical replicates were used in this assay.

Splicing misregulation is a cardinal feature of DM1, which is characteristic of an adult-to-embryonic switch in splicing patterns. The misspliced embryonic mRNA isoforms are thought unable to fulfill the adult tissue function, resulting in gradual neurodegeneration (Chau and Kalsotra, 2015). As RNA foci can be indicative of RNA binding protein sequestration and missplicing, we hypothesize that the observed reduction of RNA foci post *Lsh*Cas13a treatment could potentially translate to a reversal of splicing defects. We tested splicing patterns of six genes that are known to be regulated by MBNL1 and contribute to DM1 pathogenesis [*troponin T2* – *TNNT2*, *insulin receptor* – *INSR*, *sarcoplasmic/endoplasmic reticulum calcium ATPase 1* – *ATP2A1* or *SERCA1*, *dystonin* – *DST*, and *Muscleblind 1/2* – *MBNL 1/2* (Savkur et al., 2001; Kuyumcu-Martinez and Cooper, 2006; Hino et al., 2007; Childs-Disney et al., 2013; Santoro et al., 2013; Arandel et al., 2017; Batra et al., 2017; Renna et al., 2017, 2019)]. We observed a missplicing rescue in all six tested genes in DM1 myoblasts transduced with CUG-targeting *Lsh*Cas13a (Figure 3A and Supplementary Figure 4). However, we also observed a partial rescue with the non-targeting *Lsh*Cas13a treatment (Figure 3A). We further performed immunofluorescence (IF) against MBNL1 in normal, untreated DM1, non-targeting and CUG-targeting DM1 myoblast cells (Supplementary Figure 5) and observed a more dispersed MBNL1 distribution in *Lsh*Cas13a treated cells (Supplementary Figure 5), which may account for the splicing rescue.

### *Lsh*Cas13a Marginally Reduces *DMPK* mRNA Level in Treated Cells

To further test if the mRNA level of the repeat-residing *DMPK* gene was affected by the CUG-targeting *Lsh*Cas13a, we extracted the total RNA from normal myoblasts, DM1 myoblasts, non-targeted DM1 myoblasts, and CUG-targeting DM1 myoblasts. Quantitative RT-PCR was subsequently performed using *DMPK* primers specific to exon 9. By setting the *DMPK* mRNA level from normal myoblasts as one (Figure 3B white bar), we calculated the relative normalized *DMPK* mRNA level from each cell line. We observed a 2.9-fold increase in *DMPK* mRNA level in DM1 myoblasts (Figure 3B orange bar vs. white bar). The non-targeted DM1 myoblasts showed a 3% reduction in *DMPK* mRNA level compared to the non-treated DM1 myoblasts (Figure 3B blue bar vs. orange bar, 2.81 vs. 2.9). In contrast, the CUG-targeting DM1 myoblasts showed a 20% reduction in *DMPK* mRNA level (Figure 3B green bar vs. orange bar, 2.32 vs. 2.9). The above data suggest that the *Lsh*Cas13a treatment significantly reduced CUG RNA foci and rescued several gene missplicing defects without compromising the *DMPK* mRNA level. We have designed qRT-PCR primers to check potential off-target effects on two human RNAs that contain short CUG repeats, namely *MAP3K4* and *CASK*. Cas13a knocks down about 50% of *MAP3K4* mRNA and 30% of *CASK* mRNA in DM1 myoblasts (either non-targeting or CUG-targeting, Supplementary Figures 6A,B). Despite the off-target knockdown, the residual *MAP3K4* and *CASK* mRNAs remain within or higher than the normal range and may be sufficient to sustain normal cellular activity without yielding detrimental effects.

## Conclusion

Expansions of unstable short repeats in the human genome cause an array of neurological and neuromuscular diseases. Although symptomatically and pathologically diverse, these diseases do share an overlapping molecular mechanism, that is, the accumulation of expanded RNA. The expanded RNA can induce cellular toxicity by sequestering various RNA binding proteins as an RNA gain-of-function, or by translation into expanded proteins as a protein gain-of-function. In light of recent findings on RAN translation and the presence of RNA nuclear foci in Huntington’s disease (Zu et al., 2011; Banez-Coronel et al., 2015; Rue et al., 2016; Cleary and Ranum, 2017; Urbanek et al., 2017), the distinction between the two types of toxicity has blurred. Thus, a therapeutic strategy that can target the repeat RNA upstream of the pathogenic cascade may help transition treatment from palliative care to prevention or reversal of disease progression.

In this study, we utilized a recently discovered CRISPR-Cas13a family member *Lsh*Cas13a to track and degrade the expanded CUG repeats that cause DM1. The expanded CUG repeats are known to form RNA foci with sequestered proteins in the nucleus. Such RNA-protein rich complexes can be an ideal candidate to test the validity of using the eGFP-conjugated d*Lsh*Cas13a as an RNA tracker. However, the tendency of CUG expansion to form complex gel-like structures may pose access barriers for crRNA (Jain and Vale, 2017). Inferring from the fact that ASOs composed of 3–25 CAG repeats can bind and degrade CUG RNA foci (Mulders et al., 2009; Evers et al., 2011; Lee et al., 2012), we speculate that a repeat-based crRNA with a spacer of 28nt (annealing to approximately 9 CUG repeats) should be sufficient to guide the d*Lsh*Cas13a tracker to RNA foci. Indeed, we observed statistically significant colocalization between the tracker and CUG_x960_ repeat foci in COS-M6 cells. By designing a crRNA targeting the 3′-UTR of the *ACTB* mRNA, we were able to image stress granules in the cytoplasm. These results suggest that d*Lsh*Cas13a-eGFP is a flexible and reprogrammable RNA tracker that can reach different cellular compartments with the appropriate crRNA. A negative-feedback system using zinc finger self-targeting and KRAB domain repression on d*Lsh*Cas13a may help further reduce background noise (Abudayyeh et al., 2017).

We showed biochemically that the purified *Lsh*Cas13a cleaved more than 50% of its CUG target within 16 h in the presence of the repeat-based crRNA (Figure 2B). We attribute the relatively slow cleavage rate to the formation of multiple denaturation-resistant RNA duplexes (Supplementary Figure 3C). These RNA duplex conformations can survive 7 M urea and 30% formamide and may pose a significant energy barrier for target loading into *Lsh*Cas13a. We also observed non-specific background CUG cleavage by *Lsh*Cas13a in the absence of a crRNA (Figure 2B), which is different from the collateral RNA degradation commonly seen in the Cas13a family where a non-targeted RNA is cleaved by the activated Cas13a (Abudayyeh et al., 2016; East-Seletsky et al., 2016; Konermann et al., 2018; Myhrvold et al., 2018; Yan et al., 2018). We suggest three explanations for the non-specific RNA cleavage without a properly matched crRNA: (1) The digestion time required for CUG repeats (16 h) is much longer than that for non-repetitive targets [typically 1–2 h (Abudayyeh et al., 2016)], possibly due to repeat RNA’s complex three-dimensional folding [hairpin and gel-like nature (Tian et al., 2000; Jain and Vale, 2017)]. (2) It is evident that in the apo state *Lsh*Cas13a crystal structure a portion of the target loading channel and the two HEPN cleavage modules are located on the surface of the protein (Liu et al., 2017). The observed non-specific CUG cleavage without crRNA in this study could be due to stochastic loading of target into the apo *Lsh*Cas13a over the course of digestion. (3) Given the large size of *Lsh*Cas13a, the protein might not be stable (Supplementary Figures 3A,B); partial degradation may give rise to peptides without proper catalytic constraints. However, it is also possible that Cas13a may exhibit an intrinsic RNA cleavage activity even in the absence of crRNA-target base pairing. In parallel, dCas9 conjugated to a GFP cleaves CUG repeats as efficiently as does dCas9 conjugated to a PIN RNase domain (Batra et al., 2017), suggesting that target promiscuity might be common amongst Cas proteins. In addition, the background cleavage we observed is unlikely due to the CUG imperfect hairpin structure that may mimic the bulged 5′-crRNA handle (Napierala and Krzyzosiak, 1997; Nalavade et al., 2013), because substitutions of key residues in the 5′-crRNA hairpin result in complete abolishment of Cas13a activity (Abudayyeh et al., 2016; Liu et al., 2017).

The ability of *Lsh*Cas13a to cleave the expanded CUG repeats was further demonstrated by lentiviral delivery into patient-derived DM1 myoblast cells. By FISH we showed that > 20% of these cells contain >4 RNA foci per cell (Figure 2E). The CUG-targeting *Lsh*Cas13a reduced the population of DM1 myoblasts with >4 RNA foci per cell by approximately 20% (Figure 2E). The incomplete removal of foci was different from those observed with ASOs and PIN-conjugated dCas9 (PIN-dCas9) (Wheeler et al., 2012; Batra et al., 2017), largely due to different enzymatic mechanisms at work. ASO gapmers that form DNA-RNA hybrids elicit endogenous RNase H activity to degrade their target RNA. The PIN-dCas9 with repeat-based sgRNA (without a PAMer sequence) may operate under a complex mechanism, for: (1) The dCas9-sgRNA complex is known to bind more tightly to double-stranded DNA and DNA-RNA heteroduplex than to single-stranded RNA without a PAMer sequence (O’Connell et al., 2014). (2) The PIN domain derived from human SMG6 has high similarity of overall fold to RNase H, and the catalytic triad of acidic residues in the PIN domain are also crucial for RNase H activity (Glavan et al., 2006). Despite the incomplete foci clearance by *Lsh*Cas13a, the CUG-targeting *Lsh*Cas13a was sufficient to yield a reversal of splicing defects in six MBNL1-regulated genes that are known to contribute to DM1 phenotype (Figure 3A). The above observation raises the question as to whether a complete RNA foci elimination is required to see phenotypic improvement in cells. We are currently establishing DM1 myoblasts differentiated from induced pluripotent stem cells (iPSCs) with different repeat lengths to further validate *Lsh*Cas13a’s capacity to track and eliminate CUG foci and to rescue missplicing under varying RNA toxicity load.

Off-target effects and delivery are two major concerns of using CRISPR-Cas systems. Although crRNA could be designed to target unique sequences flanking *DMPK* CUG repeats to reduce off-target effects, it is possible that such cleavage may not extend to the repeat region and the liberated “pure” CUG repeats may still exert toxicity as shown to form gel-like structures that trap MBNL (Jain and Vale, 2017). By designing repeat-based crRNA, we initially speculate that the accumulation of CUG repeat expansions would provide more target sites for *Lsh*Cas13a to cleave, which may dissipate potential off-target effects. In line with our speculation, a small molecule with a bleomycin-cleavage module binding to every 2 CUG repeats preferentially cleaves CUG_500_ over CUG_5_ and other short CUG-containing RNA (Rzuczek et al., 2017). Furthermore, ASOs with CAG_5_, CAG_7_, or CAG_10_ silence long CUG transcripts more efficiently than short CUG transcripts in DM1 myoblasts (Gonzalez-Barriga et al., 2013). Our experiments show that the levels of *DMPK* and other CUG-containing mRNAs are affected by CUG-targeting *Lsh*Cas13a, but the residual mRNA levels are comparable to or higher than the wildtype levels. Further analyses with different DM1-relevant cell lines (e.g., cardiomyocytes and neurons), and animal models will provide additional information on phenotypic correction and off-target effects. Finally, the *Lsh*Cas13a-crRNA locus is approximately 7.8 kb in size and poses a challenge for adeno-associated viral (AAV) delivery (a packaging capacity of around 4.5 kb). In light of recent success of using a dual-vector AAV delivery system for gene editing (Bengtsson et al., 2017), it is possible to encode *Lsh*Cas13a (≈4.1 kb) and crRNA on two separate AAV vectors. Overall, our findings highlight the potential use of Cas13a to modulate the repeat RNA level. With our growing understanding of crRNA design and prediction and the choice of different Cas13 proteins at hand, the CRISPR-Cas13a strategy could potentially be extended to treating other microsatellite expansion diseases.

## Materials and Methods

### Ethics

The studies involving human participants were reviewed and approved by the Institutional Review Board of the University of Florida. The patients/participants provided their written informed consent to participate in this study.

### Plasmid Constructs

The pcDNA3.1hygro-CTGx30 plasmids used in *in vitro* transcription were cloned into the vector backbone (Thermo Fisher) with NheI and HindIII. Wildtype *Lsh*Cas13a was cloned from pC001 (Addgene #79150, gift from Feng Zhang) into pAcGFP-C1 (Clonetech) and subsequently underwent site-directed mutagenesis to create the pAcGFP-C1-*Lsh*C2c2R1278A plasmid. To construct the tracker vector, the pX330-U6-Chimeric_BB-CBh-hSpCas9 plasmid (Addgene #42230, gift from Feng Zhang) was modified through the following steps: (1) Replacing the pU6-Chimeric gRNA scaffold with pU6-DR-crRNA containing two inverted BsaI sites (IDT). The BsaI sites were designed for spacer insertion. (2) Replacing the CBh promoter with pCMV from pAcGFP-C1. (3) Primer walk to concatenate nucleoplasmin NLS, *Lsh*Cas13aR1278A, linker, eGFP (from the pC015-dLwCas13a-NF plasmid, Addgene #91905, gift from Feng Zhang) and c-Myc NLS. To construct the lentiviral expression vector, the pU6-sgRNA scaffold sequence was replaced with pU6-crRNA (IDT) in the lentiCRISPR v2 plasmid (Addgene #52961, gift from Feng Zhang). Subsequently, the *Sp*Cas9 sequence was replaced with the concatenated *Lsh*Cas13a-nucleoplasmin NLS-SV40 NLS-P2A-pyromycin resistant gene.

### Cell Culture and Transfection

COS-M6 cells (an African green monkey kidney cell line) were commercially obtained and cultured in DMEM with 10% FBS and 1% penicillin/streptomycin (GIBCO) and passaged at 80–90% confluency. HEK293FT cells were cultured in GlutaMAX supplemented with 10% FBS and 1% penicillin/streptomycin and passaged at 80–90% confluency. Myoblasts were cultured in skeletal muscle growth medium (Cell Applications) and passaged at 70–80% confluency. COS-M6 cells were seeded at 5x10^5^ cells per well of a 6-well plate the day before transfection. For FISH studies, 2 μg of DT960 and 2 μg of the tracker plasmid were mixed with 10 μl of Lipofectamine 2000 (Thermo Fisher) in OptiMem during transfection. For FISH-IF studies, 2 μg of the *ACTB* tracker vector at a 1:5 ratio to Lipofectamine 2,000 were used.

### Lentiviral Production and Transduction

LentiCRISPR v2-derived constructs encoding d*Lsh*Cas13a and crRNA were co-transfected with psPAX2 (Addgene #12260, packaging vector, gift from Didier Trono) and pMD2.G (Addgene #12259, envelop vector, gift from Didier Trono) into HEK293FT cells at a ratio of 3.3:2.5:1 (a total of 17 μg DNA) on a 10 cm plate with Fugene 6 (Promega). Viruses produced at 48 and 72 h were filtered with 0.45 μm PVDF filter units (Millex-HV), checked for titer with Lenti-X GoStix (Takara), and used for transduction on myoblasts in the presence of 8 μg/ml polybrene per well of a 6-well plate. Cells were selected in 2 μg/ml puromycin for 5 days.

### RNA Fluorescence *in situ* Hybridization

Experiments were conducted as described in Manning et al. (2017). Twenty-four hours post transfection, cells on round coverslips were fixed in 4% PFA in PBS for 15 min, permeabilized in 0.2% Triton X-100 in PBS for 10 min, and incubated in 40% formamide/2x SSC for 10 min. Cells were then incubated with 200 nM Cy3-labeled PNA probes (PNA Bio) in the hybridization buffer (40% formamide/2x SSC/0.02% BSA) at 37°C for 1 h and washed with 40% formamide/1x SSC at 37°C for 1 h, twice with 1x SSC for 30 min, and four times with PBS (5 min each) before mounted in mounting medium with DAPI (Vectorlabs). Images were taken at x60 objective on a Nikon A1 Confocal Imaging System with filters specifically for Cy3 and eGFP under a sequential stimulation mode. For quantification of RNA foci in each cell line, data were collected from two independent experiments each with two technical replicates. All images within each experiment were taken under identical exposure and transmittance.

### Immunofluorescence

To induce stress granule formation, 200 μM sodium arsenite were added per well of a 6-well plate at 37°C for 1 h 30 min. After 1x PBS wash, cells were fixed in 4% PFA in PBS for 15 min, permeabilized in 0.2% Triton X-100 in PBS for 10 min, and blocked in 5% BSA in PBST for 1 h. Mouse monoclonal anti-G3BP1-Alexa 594 (H-10, Santa Cruz, CA, United States) antibody was added to cells at 4 μg/ml in 5% BSA-PBST at 4°C overnight. After four PBS washes (5 min each), cells were mounted in mounting medium containing DAPI for imaging. Two biological samples each with two technical replicates were imaged. The mouse monoclonal anti-MBNL1 antibody (Sigma M3320, clone number 3A4-1E9, immunogen – recombinant MBNL1) was used for IF.

### *Lsh*Cas13a Protein Expression and Purification

*Lsh*Cas13a was expressed from the pC001-huLshC2c2-MBP vector (gift from Feng Zhang) in *E. coli* BL21 (DE3) cells. *E. coli* cells were grown in 1 L LB broth (Sigma) at 37°C to reach OD_600_ = 0.4–0.7 before induction with 0.5 mM IPTG at 18°C overnight. Cells were harvested by centrifugation at 5,000 rpm, resuspended in lysis buffer (20 mM Tris–HCl pH7.5/500 mM NaCl/5% glycerol), and frozen at −80°C overnight.

Frozen cells were thawed, sonicated on ice for two 10 min cycles of 40% amplitude 2 s on-off, and centrifuged at 14,000 rpm to obtain the soluble fraction. The soluble lysate was incubated with NiNTA agarose beads (Qiagen) of a 5 mL bed volume, washed with 7 volumes of wash buffer (50 mM Tris–HCl pH7.5/2 M NaCl/5 mM MgCl2/5% glycerol), eluted under a linear gradient of elution buffer (50 mM Tris–HCl pH7.5/2 M NaCl/5 mM MgCl2/1 M imidazole/5% glycerol), and dialyzed in 50 mM Tris–HCl pH7.5/500 mM NaCl/5 mM MgCl2/1 mM DTT/5% glycerol. The dialyzed *Lsh*Cas13a was further purified with amylose agarose (NEB) of a 3 ml bed volume with wash buffer (20 mM Tris–HCl pH7.5/1 M NaCl/5% glycerol) and elution buffer (20 mM Tris–HCl pH7.5/1 M NaCl/10 mM Maltose/5% glycerol). The (His)_x6_-MBP tag was removed by thrombin (EMD Millipore) at 4°C overnight. The tag, uncleaved *Lsh*Cas13a and biotinylated thrombin were captured by a mixture of streptavidin and NiNTA agarose beads in column. The flow-through that contains the purified protein was dialyzed in 50 mM Tris–HCl pH7.5/500 mM NaCl/1 mM DTT/5% glycerol.

### Generation of RNA

The pcDNA3.1 hygro-CTGx30 plasmids were linearized and gel purified. Approximately 500 ng of DNA template were used to synthesize RNA repeats at 30°C overnight using the HiScribe T7 High Yield Kit (NEB) according to the manufacturer’s protocol. For crRNA synthesis, a synthetic short T7 primer was annealed to the synthetic crRNA (both oligos were ordered from IDT, Oligonucleotide list, Supplementary Table 1) in the annealing buffer (30 mM HEPES pH7.5/100 mM potassium acetate/2 mM magnesium acetate) at 95°C for 5 min, followed by a gradual cool-down to room temperature. All the transcribed RNA underwent Turbo DNase I (Thermo Fisher) digestion and purified with RNA Clean & Concentrator (Zymo Research).

### *Lsh*Cas13a *in vitro* Cleavage Assay

The cleavage assays were performed in 10 μl volumes, containing: nuclease buffer (40 mM Tris–HCl pH7.5/60 mM NaCl/6 mM MgCl2), 450 nM Hu*Lsh*Cas13a, 100 nM target RNA and 100 nM crRNA. The crRNA was pre-loaded with *Lsh*Cas13a at 37°C for 15 min before the target RNA was added. Reactions were allowed to proceed over a period of time (0–16 h), quenched with 2x TBE-Urea loading dye (BioRad) mixed with 3x Formamide dye (100% formamide/0.5 M EDTA), boiled at 95°C for 5 min, and loaded on a 10% TBE-Urea gel. Gels were stained with SYBR Gold (Thermo Fisher) at a 1:10,000 dilution for 20 min before imaged using an Azure c400 scanner (Azure Biosystems).

### qRT-PCR and Missplicing Assays

Total RNA extractions on 10 cm plates were performed with RNeasy Mini Kit (Qiagen), followed by Turbo DNase I digestion and column purification. PCR reactions were performed on purified RNA with β-actin primers to ensure the absence of genomic DNA contamination for each sample. A 1 μg input of total RNA was converted to cDNA using the iScript cDNA Synthesis Kit (BioRad) according to the manufacturer’s protocol. Typically, 2.5 μl of the diluted cDNA (1:3 dilution) were used for qRT-PCR on a CFX96 Real-time System (BioRad). Three biological replicates, each with three technical replicates, were performed.

For the missplicing assay, 2.5 μl of the diluted cDNA were incubated with Amplitaq Gold 360 Master Mix (Thermo Fisher) with 95°C 5 min, 35x (95°C 30 s, 61°C 30 s and 72°C 1 min), 72°C 5 min and 4°C hold. Reactions were resolved on a 2% agarose gel, scanned using Azure c400, and quantified using Fiji ImagJ. Two independent experiments were performed for each sample.

### Quantification and Statistical Analysis

RNA foci counts were performed in Fiji ImageJ with the “Find Maxima” function at a threshold level of 80. Significance levels were calculated using Chi-square with 4 foci per cell as a cut-off.

Colocalization between stress granules and the d*Lsh*Cas13a tracker was analyzed in Fiji ImageJ. Images were processed with “Split Channels.” Each cell was applied with an ROI mask before analyzed by “Coloc 2” to get the Pearson’s R value. R values were tabulated and two-tailed *t*-tests were performed in GraphPad Prism 7. Statistical values used: ^∗^*P* < 0.05, ^∗∗^*P* < 0.01, and ^∗∗∗^*P* < 0.001.

#### *In vitro* RNA Cleavage and Splicing Were Quantified Using Fiji ImageJ

All qRT-PCR data were analyzed in BioRad CFX Manager. Two-tailed *t*-tests were performed in GraphPad Prism 7. Statistical values used: ^∗^*P* < 0.05, ^∗∗^*P* < 0.01, and ^∗∗∗^*P* < 0.001.

## Data Availability Statement

The raw data supporting the conclusions of this article will be made available by the authors, without undue reservation.

## Author Contributions

NZ and BB carried out cloning. NZ expressed and purified *Lsh*Cas13a and performed biochemical cleavage assays, performed FISH, FISH/IF, missplicing assays and qRT-PCR, and devised the experiments. GX and DF provided critical experimental cell lines. NZ and TA performed data analysis and wrote the manuscript. All authors contributed to the article and approved the submitted version.

## Conflict of Interest

The authors declare that the research was conducted in the absence of any commercial or financial relationships that could be construed as a potential conflict of interest.

## Footnotes

1 https://www.myotonic.org/ionis-dmpkrx
