# Supplementary material for: A CRISPR-Cas13a Based Strategy That Tracks and Degrades Toxic RNA in Myotonic Dystrophy Type 1
Source: Front Genet. 2020 Dec 10;11:594576. doi: 10.3389/fgene.2020.594576 (PMC7758406; doi:10.3389/fgene.2020.594576)
Supplement: Supplementary file 1 [file Data_Sheet_1.docx]

**Supplementary information**


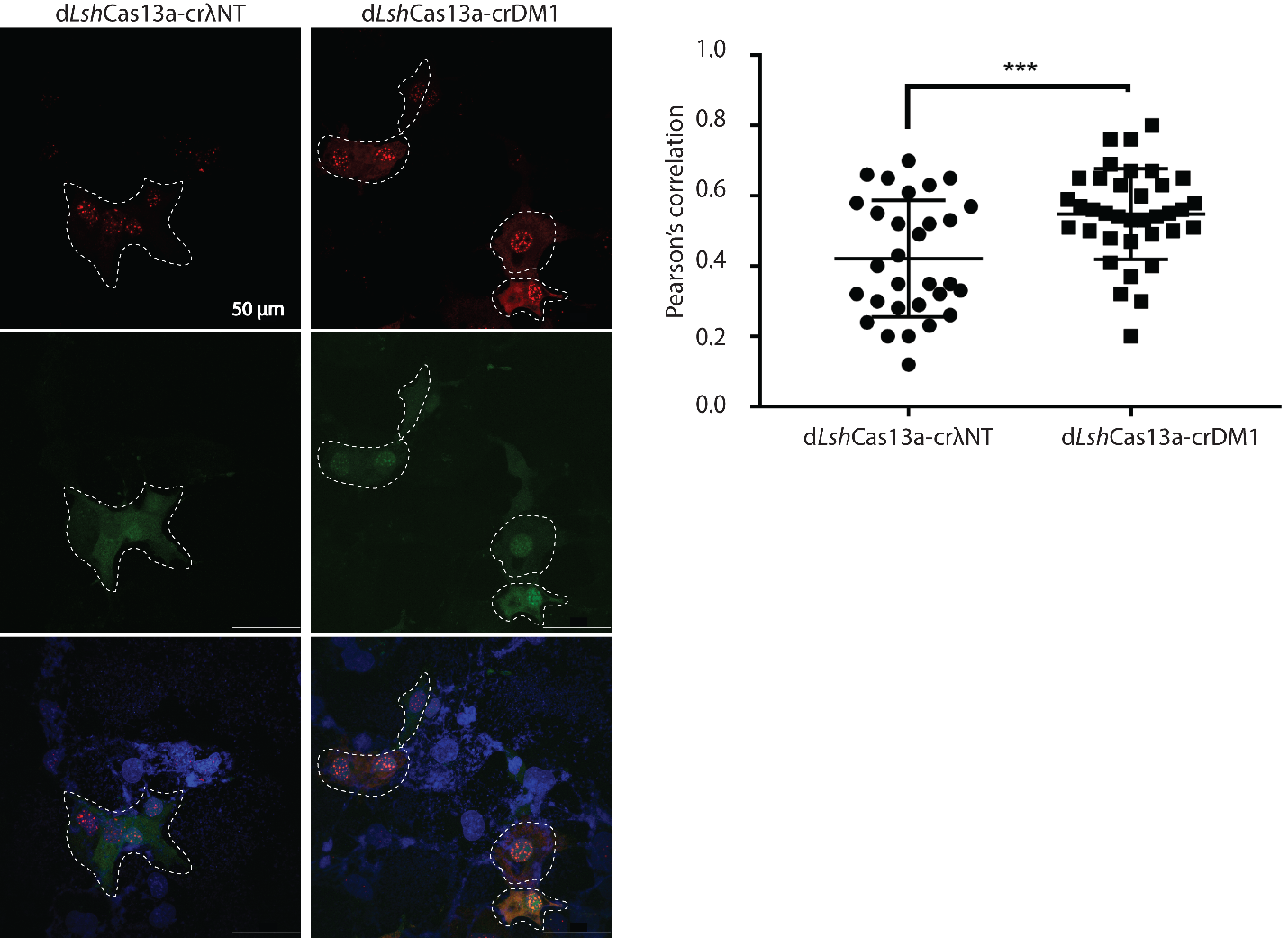


**Fig. S1. Statistical analysis of colocalization between the *Lsh*Cas13a tracker and FISH-detected CUG RNA foci in COS-M6 cells.** Representative cells were contoured with dotted white lines as regions of interest (ROI). Each ROI was used as a mask in both green and red channels to generate Pearson’s correlation under the “Coloc 2” function in Fiji ImageJ. Pearson’s correlation was tabulated in GraphPad Prism 7 for unpaired t-test (p = 0.0009, *** p = 0.001).


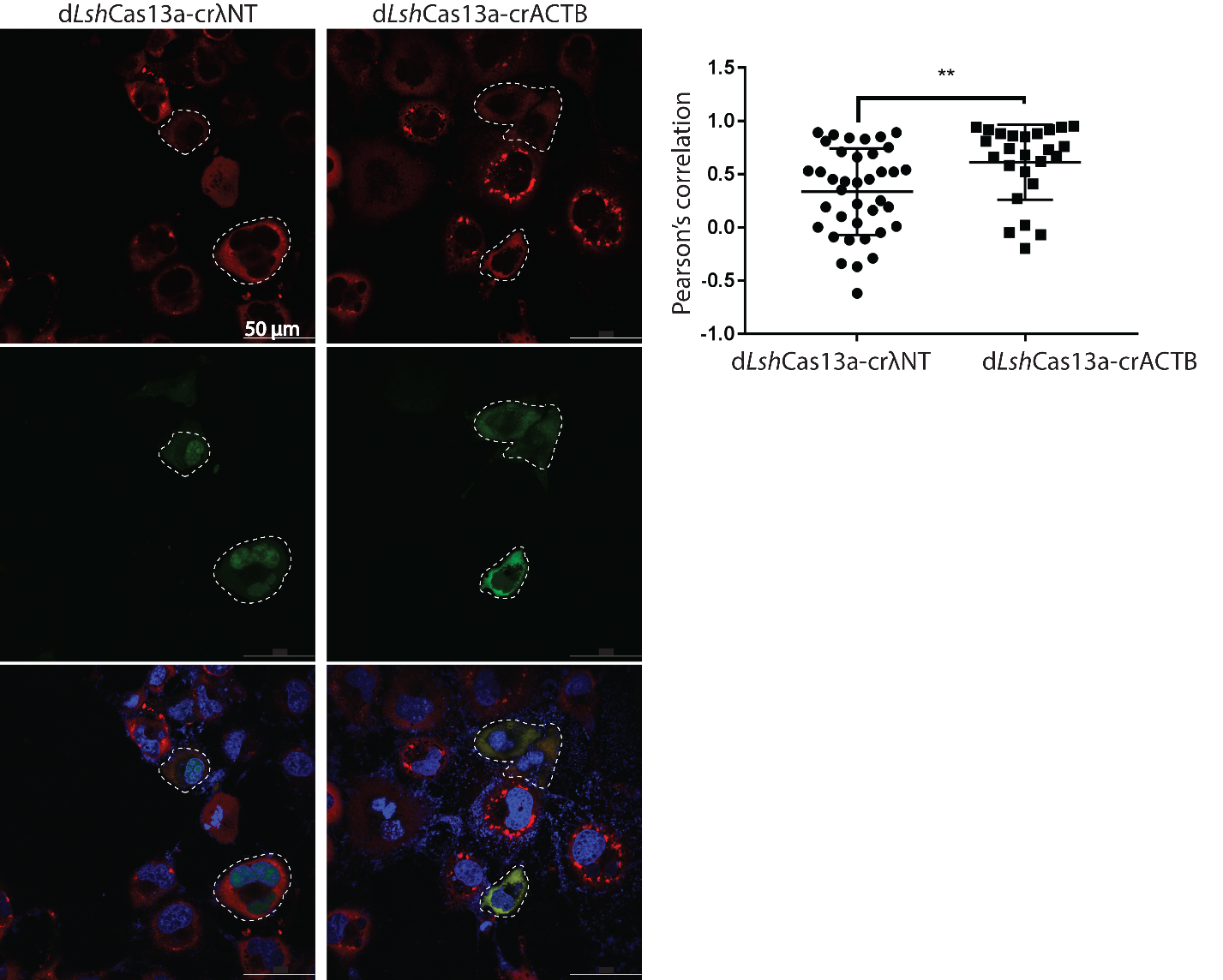


**Fig. S2.** **Statistical analysis of colocalization between the *Lsh*Cas13a tracker and anti-G3BP1-labelled stress granules in COS-M6 cells.** Representative cells were contoured with dotted white lines as regions of interest (ROI). Each ROI was used as a mask in both green and red channels to generate Pearson’s correlation under the “Coloc 2” function in Fiji ImageJ. Pearson’s correlation was tabulated in GraphPad Prism 7 for unpaired t-test (p = 0.0069, ** p = 0.01).


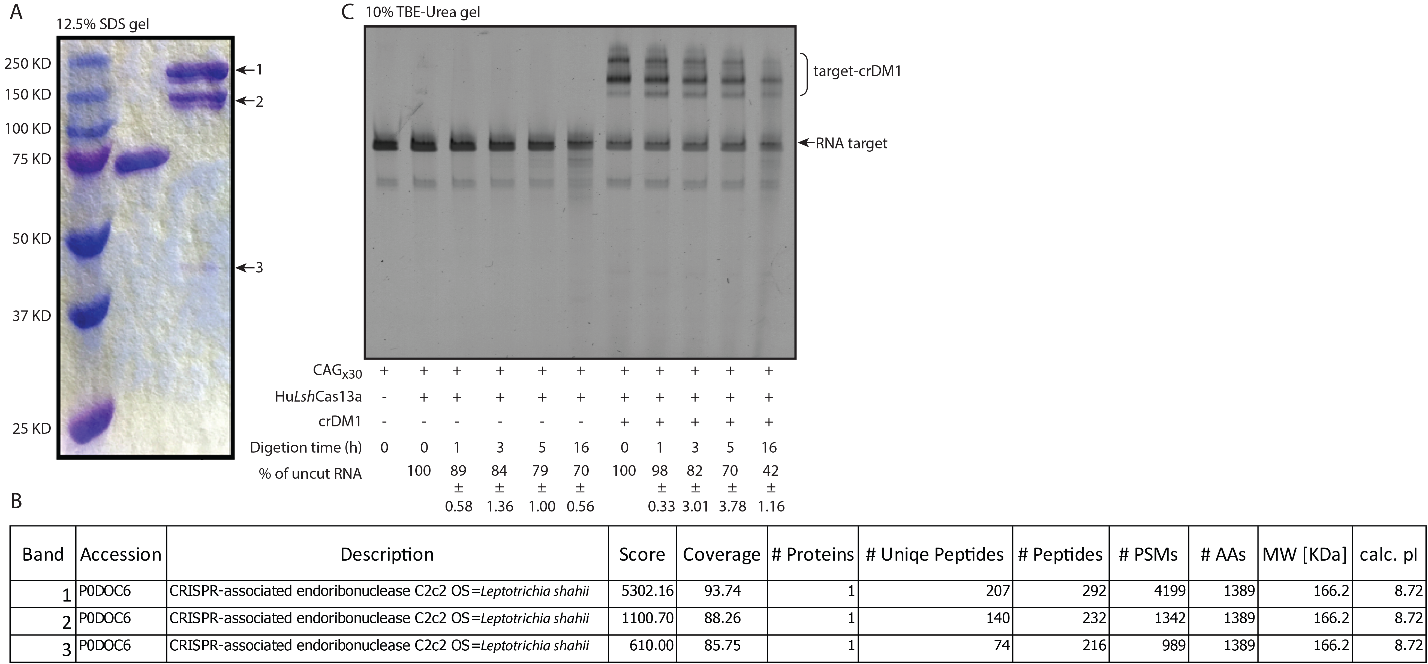


**Fig. S3. Purification of *Lsh*Cas13a and *in vitro* cleavage of CUG_x30_ repeats.** (A) The *Lsh*Cas13a protein was purified from *E. coli* through chromatography. Ten microliters of 2 µM BSA and *Lsh*Cas13a were each loaded on a 12.5% SDS-PAGE gel to check for purity. Two additional bands were observed in the purified *Lsh*Cas13a (arrows 2 and 3). These bands were excised from the gel, analyzed by mass spectroscope, and identified as truncated *Lsh*Cas13a (B). (C) Cleavage of CUG_x30_ RNA repeats by purified *Lsh*Cas13a and *in vitro* transcribed crDM1.


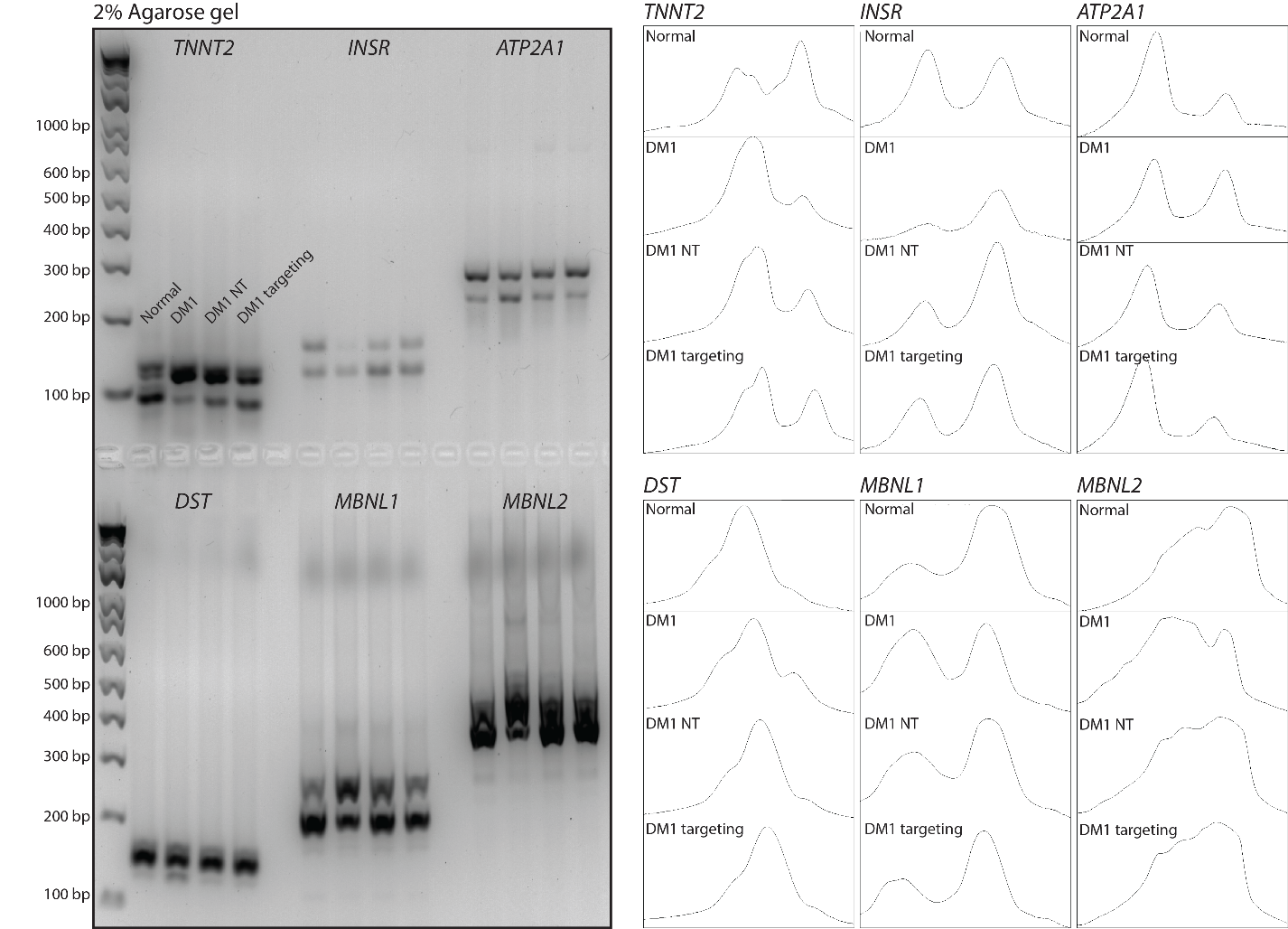


**Fig. S4. Uncropped gel of missplicing rescue by *Lsh*Cas13a transduced into DM1 myoblast cells.**


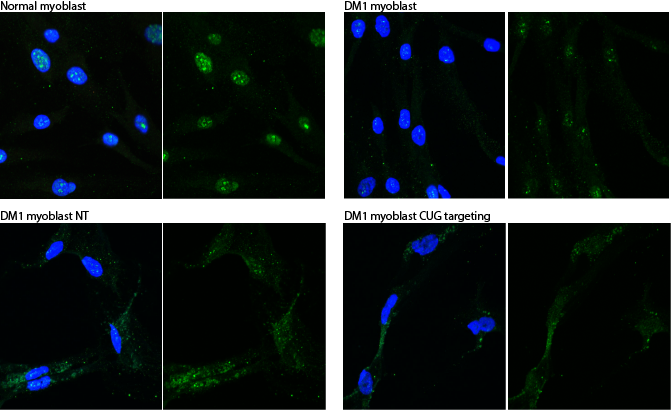


**Fig. S5. Distribution of MBNL1 in normal, untreated DM1, non-targeting and CUG-targeting DM1 myoblasts.** IF was performed with anti-MBNL1 antibodies (Sigma clone 3320) at 1:400.


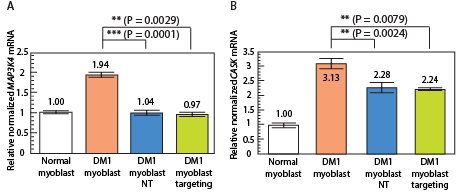


Fig. S6. Off-target cleavage by CUG-targeting *Lsh*Cas13a on *MAP3K4* and *CASK* mRNAs. Two-tailed t-tests were calculated in GraphPad Prism 7 (** p = 0.01, *** p = 0.001).

**Table S1. List of oligonucleotides used in this study**
